# Improved Nutritive Quality and Salt Resistance in Transgenic Maize by Simultaneously Overexpression of a Natural Lysine-Rich Protein Gene, *SBgLR*, and an ERF Transcription Factor Gene, *TSRF1*

**DOI:** 10.3390/ijms14059459

**Published:** 2013-04-29

**Authors:** Meizhen Wang, Chen Liu, Shixue Li, Dengyun Zhu, Qian Zhao, Jingjuan Yu

**Affiliations:** 1 State Key Laboratory of Agrobiotechnology, College of Biological Sciences, China Agricultural University, No. 2 Yuanmingyuan West Road, Haidian District, Beijing 100193, China; E-Mails: mzwang1981@yahoo.com.cn (M.W.); liuchensh@yahoo.com.cn (C.L.); lishixue19890105@126.com (S.L.); zhudy@cau.edu.cn (D.Z.); zhaoqian@cau.edu.cn (Q.Z.); 2 Institute of Medicinal Plant Development, Chinese Academy of Medical Sciences & Peking Union Medical College, No. 151, Malianwa North Road, Haidian District, Beijing 100193, China

**Keywords:** *Zea may* L., high lysine, high protein, salt tolerance, marker-free

## Abstract

Maize (*Zea mays* L.), as one of the most important crops in the world, is deficient in lysine and tryptophan. Environmental conditions greatly impact plant growth, development and productivity. In this study, we used particle bombardment mediated co-transformation to obtain marker-free transgenic maize inbred X178 lines harboring a lysine-rich protein gene *SBgLR* from potato and an ethylene responsive factor (ERF) transcription factor gene, *TSRF1*, from tomato. Both of the target genes were successfully expressed and showed various expression levels in different transgenic lines. Analysis showed that the protein and lysine content in T_1_ transgenic maize seeds increased significantly. Compared to non-transformed maize, the protein and lysine content increased by 7.7% to 24.38% and 8.70% to 30.43%, respectively. Moreover, transgenic maize exhibited more tolerance to salt stress. When treated with 200 mM NaCl for 48 h, both non-transformed and transgenic plant leaves displayed wilting and losing green symptoms and dramatic increase of the free proline contents. However, the degree of control seedlings was much more serious than that of transgenic lines and much more increases of the free proline contents in the transgenic lines than that in the control seedlings were observed. Meanwhile, lower extent decreases of the chlorophyll contents were detected in the transgenic seedlings. Quantitative RT-PCR was performed to analyze the expression of ten stress-related genes, including stress responsive transcription factor genes, *ZmMYB59* and *ZmMYC1*, proline synthesis related genes, *ZmP5CS1* and *ZmP5CS2*, photosynthesis-related genes, *ZmELIP*, *ZmPSI-N*, *ZmOEE*, *Zmrbcs* and *ZmPLAS*, and one ABA biosynthesis related gene, *ZmSDR*. The results showed that with the exception of *ZmP5CS1* and *ZmP5CS2* in line 9–10 and 19–11, *ZmMYC1* in line 19–11 and *ZmSDR* in line 19–11, the expression of other stress-related genes were inhibited in transgenic lines under normal conditions. After salt treatment, the expressions of the ten stress-related genes were significantly induced in both wild-type (WT) and transgenic lines. However, compared to WT, the increases of *ZmP5CS1* in all these three transgenic lines and *ZmP5CS2* in line 9–10 were less than WT plants. This study provides an effective approach of maize genetic engineering for improved nutritive quality and salt tolerance.

## 1. Introduction

Maize (*Zea mays* L.), also known as corn, is one of the most widely cultivated crops in the world. It is mainly used as human food, livestock feed and industrial raw material. However, malnutrition is common in the countries, where corn is the sole or primary food source, due to the deficiency of essential amino acids, like lysine and tryptophan [1]. Actually, major efforts have been made to identify high-lysine corn varieties by genetic approaches since the mid-twentieth century. *Opaque 2* (*o2*) is a natural high-lysine mutant, which has twice of the lysine content than in the normal maize [2]. However, several drawbacks were observed in this mutant, such as soft chalky endosperm [2]. Even though quality protein maize (QPM) has been produced by backcrossing of *o2* modifier genes into *o2* mutant [3], it is a lengthy progress (~30 years) that limits the spread of QPM. Genetic engineering technologies are also utilized aiming to increase lysine content by zein reduction [4–6]. Unfortunately, all these transgenic plants exhibit opaque kernel phenotypes, which were very similar to that of *o2* mutant [4–6]. Moreover, increase of lysine content in corn grain has been achieved by manipulation of both lysine biosynthesis and metabolic pathways [7,8], including the commercialized high-lysine maize variety LY038 [9]. However, only free lysine content is significantly increased in these transgenic plants [8].

Recently, seed-specific expression of natural proteins with high-lysine concentration has been proved to be an effective approach to increase the lysine content of corn grain. Among them, milk proteins are attractive choice due to their balanced amino acid composition and good digestibility. As reported, lysine content is enhanced obviously in maize endosperm when expressing the milk protein, α-lactalbumin [10,11], whereas total protein content is not significantly different from negative kernels [11]. Liu *et al*. (1997) cloned a pollen-specific cDNA encoding a lysine-rich protein SB401 from potato [12]. In our previous studies, seed-specific expression of *SB401* increases lysine content from 16.1% to 54.8%, and meanwhile, the total protein content is increased from 11.6% to 39.0% in T_1_ transgenic maize seeds, compared with the non-transgenic lines [13]. Thereafter, the natural lysine-rich protein gene *SBgLR*, homologous to *SB401*, was isolated from potato and transformed into maize, resulting in greater lysine and protein contents increase in transgenic maize [14]. It provides an effective way for maize quality improvement.

Environmental conditions, such as drought, salinity, extreme temperatures and pathogen attack, greatly affect plant growth, development and productivity. To survive under these conditions, plants have developed a series of defense responsive pathways [15]. In this signaling network, transcription factors play important roles through regulating expression of stress-related genes [16,17]. Ethylene responsive factor (ERF) proteins are a class of plant specific transcription factors, which have been reported to modulate plant development and multiple stress responses [18–22]. It was found that ERF proteins could function not only in plant pathogen resistance by interacting with GCC-box (AGCCGCC) [23–25], but also in abiotic resistance through interacting with the DRE element (CCGAC) [18,21,26–28]. TSRF1, an ERF protein from tomato, can activate the expression of PR genes by binding to the GCC-box and positively regulates pathogen resistance in transgenic tomato and tobacco [29,30]. ABA enhances the binding of TSRF1 to GCC-box and modifies the pathogen resistance of transgenic tobacco [31]. In addition, TSRF1 negatively regulates the osmotic response in transgenic tobacco [30], but improves the osmotic and drought tolerance in transgenic rice, indicating different mechanisms of TSRF1 in dicots and monocots [32]. All these results suggest that genetic engineering of ERF transcription factor genes is an effective way to improve plant stress tolerance [33].

Genetically modified (GM) crops are the fast-adopted technology in agriculture [34]. It has increased to 29 countries growing GM crops in recent years [35]. However, intense debates have been addressed regarding the safety of GM crops to the environment and customers. Selectable marker genes, like herbicide or antibiotic resistance genes, are supposed to be dangerous, although there is no strong scientific evidence [36]. By far, various strategies to eliminate the selectable marker genes have been developed and successfully used, such as co-transformation, site-specific recombination, multi-autotransformation vector, transposition system and homologous recombination (see more in review [35]). For *Agrobacterium*-mediated co-transformation, there are three methods: (i) two different vectors carried by different *Agrobacterium* strains [37]; (ii) two different vectors in the same *Agrobacterium* strain [38] and (iii) one binary vector with twin T-DNAs [39]. For biolistic bombardment mediated co-transformation, two different plasmids were introduced into the same tissue [40,41]. Among them, co-transformation is widely used due to its simplicity. It can be carried out either by *Agrobacterium*-mediated transformation or by biolistic bombardment. Selectable marker genes and target genes are usually integrated into the separate loci and segregate independently in the subsequent generations [42–44].

In this study, two plasmids containing the target genes, a lysine-rich protein gene, *SBgLR*, and an ERF transcription factor gene, *TSRF1*, and the selectable marker gene, *Hpt*, respectively, were introduced into maize elite inbred X178 embryogenic calli by particle bombardment. Marker-free transgenic maize lines were obtained. Further analysis revealed that both of the target genes were successfully expressed. Further, the lysine and protein content of transgenic maize seeds were detected and the stress tolerance was analyzed by measuring the free proline and chlorophyll contents. The results showed that transgenic lines had not only increased lysine and protein content, but also more tolerance to salt stress. Moreover, ten stress-related gene expression changes were analyzed using quantitative RT-PCR. Marker-free transgenic maize lines with high quality and stress resistance obtained in this research will be good materials for corn breeding.

## 2. Results and Discussion

### 2.1. TSRF1, but Not SBgLR Protein Has Homologs in Maize

**Figure 1:**
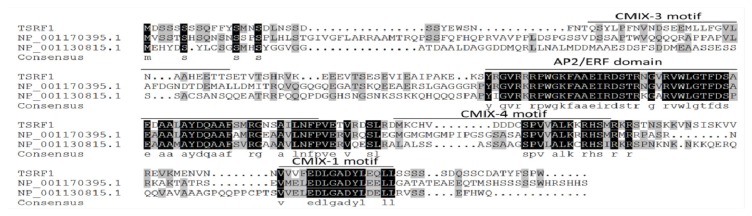
Comparison of the putative motifs of *TSRF1* and its homologs in maize. Motifs of TSRF1 were marked by black lines on the top of the sequences.

To obtain marker-free transgenic maize inbred lines, immature embryogenic calli were transformed by particle bombardment-mediated co-transformation. In this system, two constructs pTSSB and pHpt were used. As is shown in Figure S1, pTSSB contains a potato lysine-rich protein gene, *SBgLR*, under the control of seed-specific promoter, 19Z, and a tomato ERF transcription factor gene, *TSRF1*, under the control of 35S constitutive promoter. The vector pHpt contains a selectable marker gene, *Hpt*, for hygromycin selection. By searching SBgLR protein sequence against the maize reference genome using the tBLASTn program [45,46], no homologs of SBgLR gene were identified (data not shown). However, two homologs for TSRF1 gene were found by similar method (Figure 1). The TSRF1 contains four motifs: an AP2/ERF domain, a CMIX-1 motif, a CMIX-3 motif and a CMIX-4 motif [32]. No ERF proteins containing all the four motifs of TSRF1 have been identified in the japonica rice genome by bioinformatic analysis [47]. To compare the structure of TSRF1 with its homologs in maize, we performed a multiple alignment using Clustal W (http://www.ebi.ac.uk/Tools/clustalw2/index.html) [48]. In contrast to rice, the homologs of maize contain all the four conserved motifs (Figure 1). Therefore, we designed the specific primer pairs in the non-conserved region of *TSRF1* for subsequent PCR and RT-PCR detection of transgenic lines (Table S1).

### 2.2. Generation of Transgenic Maize Inbred Lines

For co-transformation, two constructs pTSSB and pHpt mixture at a mole ratio of 1.5:1 were co-bombarded into maize embryogenic calli. Different stages of transformation were shown in Figure S2A–F. A total of 114 fertile plants were obtained and self-pollinated for seeds set. In order to confirm the integration of transgenes, we conducted PCR analysis for *SBgLR*, *TSRF1* and *Hpt* genes. Partial results were shown in Figure S2G,H. Twenty-six transgenic lines positive for all three genes were identified. Transformation efficiency of 1.08% in this study was obtained (Table 1). Both target genes tend to insert into the same loci of one transgenic line. A similar result was reported in maize that an entire 10-member kafirin gene cluster was transformed into maize genome by particle bombardment method without gene silencing [49].

### 2.3. Overexpression of SBgLR and TSRF1 in Transgenic Maize

**Figure 2:**
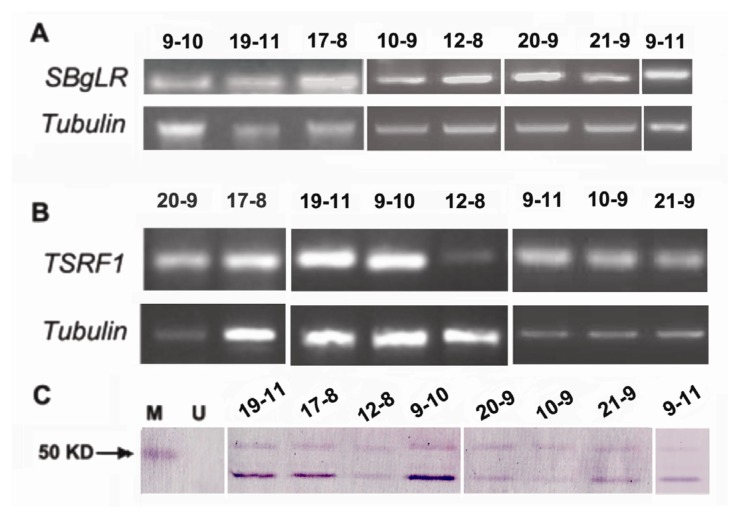
Expression of transgenes in T_1_ maize (partial results are shown). (**A**) Semi-quantification RT-PCR analysis of *SBgLR* in transgenic maize immature seeds (22 DAP); (**B**) semi-quantification RT-PCR analysis of *TSRF1* in transgenic maize leaves; (**C**) Western blot analysis of SBgLR in transgenic maize immature seeds (20 DAP). M, protein marker; U, non-transformed maize plants. Numbers on the top of each figure represent the transgenic line number.

To test whether *SBgLR* and *TSRF1* expressed in transgenic maize, we conducted semi-quantitative RT-PCR using cDNAs from T_1_ transgenic maize immature seeds at 22 days after pollination (DAP) and leaves as templates, respectively (Figure 2A,B). The results revealed that both *SBgLR* and *TSRF1* were expressed in the progeny from 16 transgenic lines, and they showed various expression levels among different transgenic lines.

Water-soluble proteins were extracted from the immature seeds (20 DAP) of T_1_ generation of *SBgLR* and *TSRF1* RT-PCR positive lines. Western blot was further carried out to confirm SBgLR protein accumulation in transgenic maize seeds (Figure 2C). Specific rabbit polyclonal antiserum against SBgLR at 1:400 was used. The predicted molecular mass of the SBgLR was 23 kD, whereas it was much larger (about 50 kD) after SDS/PAGE separation. This discrepancy was also observed in previous Western blot analysis of SBgLR and SB401 in transgenic maize [13,14]. Studies have shown that SB401 is a microtubule-associated protein and has a reduced mobility on SDS/PAGE [13,50], which is a common feature of microtubule-associated proteins [51,52]. In addition, it should be noted that two specific bands were observed in Western blot analysis of SBgLR (Figure 2C). The glycosylation site existed in the *N*-terminal of SBgLR may result in the reduction of mobility. However, further confirmation is needed. The transgenic lines with relative high *SBgLR* and *TSRF1* expression levels were selected for subsequent nutritive quality and stress tolerance analysis.

### 2.4. Marker-Free Transgenic Lines Obtained by Segregation in T_2_ Generation

**Figure 3:**
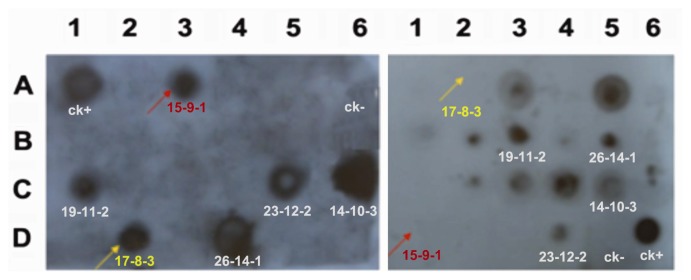
Segregation of target genes and selectable marker gene in T_2_ transgenic plants by DNA dot blot analysis. Two segregated transgenic lines are highlighted in red for 15-9-1 and yellow for 17-8-3, respectively. Transgenic lines that have only *Hpt* are not indicated. For *SBgLR* (left), A1 represents positive control and A6 represents negative control. For *Hpt* (right), D6 represents positive control and D5 represents negative control.

In the particle bombardment-mediated co-transformation system, marker gene elimination is dependent on the segregation of the target gene and selectable marker gene in the progeny. To examine whether the selectable marker gene *Hpt* was removed successfully, we performed genomic DNA dot blot to analyze T_2_ transgenic lines (Figure 3). Purified PCR fragments of *SBgLR* and *Hpt* were used as probes, respectively. The results indicated that segregation of target genes and *Hpt* occurred in T_2_ generation. Two transgenic lines 17 and 15 in R_0_ generation contained both target genes and selectable marker gene, but their corresponding T_2_ generation transgenic lines 17-8-3 and 15-9-1 harbored only target genes as highlighted by arrows. These results indicated that it is a feasible approach to obtain marker-free transgenic lines via particle bombardment mediated co-transformation.

### 2.5. SBgLR Enhanced Crude Protein and Lysine Contents in Transgenic Maize

**Figure 4:**
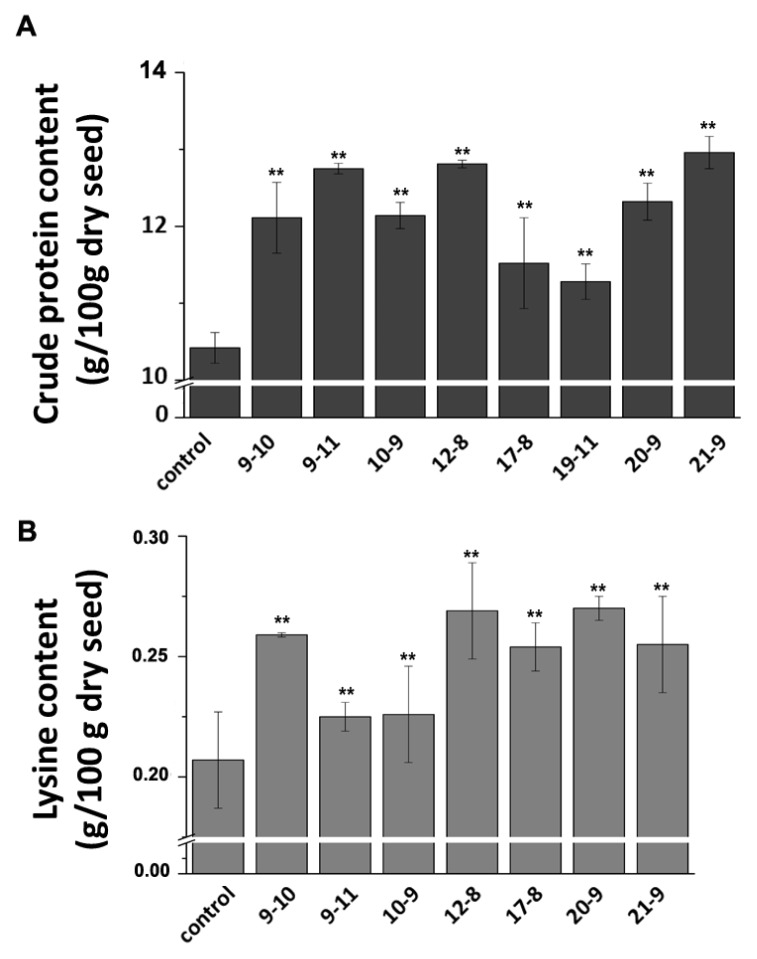
Protein and lysine levels were simultaneously increased in T_1_ transgenic seeds. (**A**) Protein content; (**B**) lysine content. The crude protein and lysine contents are expressed as g/100 g dry seed. Data are averages of triplicates ± standard deviations. Asterisks, **, denote transgenic lines statistically different from Control by Student *t*-test at *p* < 0.01. The lysine content of line 19–11 was not measured due to limited amount of seeds.

Twenty kernels from each of the self-pollinated transgenic and non-transformed T_1_ plants were randomly selected and the lysine and protein contents were analyzed. The results showed that the lysine and protein contents in the seeds were increased at various levels in different transgenic maize lines. Compared to non-transformed maize, the increases of protein and lysine content ranged from 7.7% to 24.38% (Figure 4A) and from 8.70% to 30.43% (Figure 4B), respectively. Line 19–11 showed high SBgLR accumulation level (Figure 2C) and total protein content, but its lysine content was not analyzed, due to limited amount of seeds. Subsequently, we detected the lysine content of T_2_ transgenic seeds (Table S2). The result showed that the lysine contents in all these transgenic lines were increased. In addition, it should be noted that the lysine contents in T_2_ transgenic lines were increased to a much greater extent than in T_1_ generation, indicating much more protein and lysine accumulation, because the *SBgLR* gene became homozygous. However, more evidence is needed. These transgenics will be good materials in quality maize breeding programs.

### 2.6. TSRF1 Increased Salt Tolerance in Transgenic Maize

**Figure 5:**
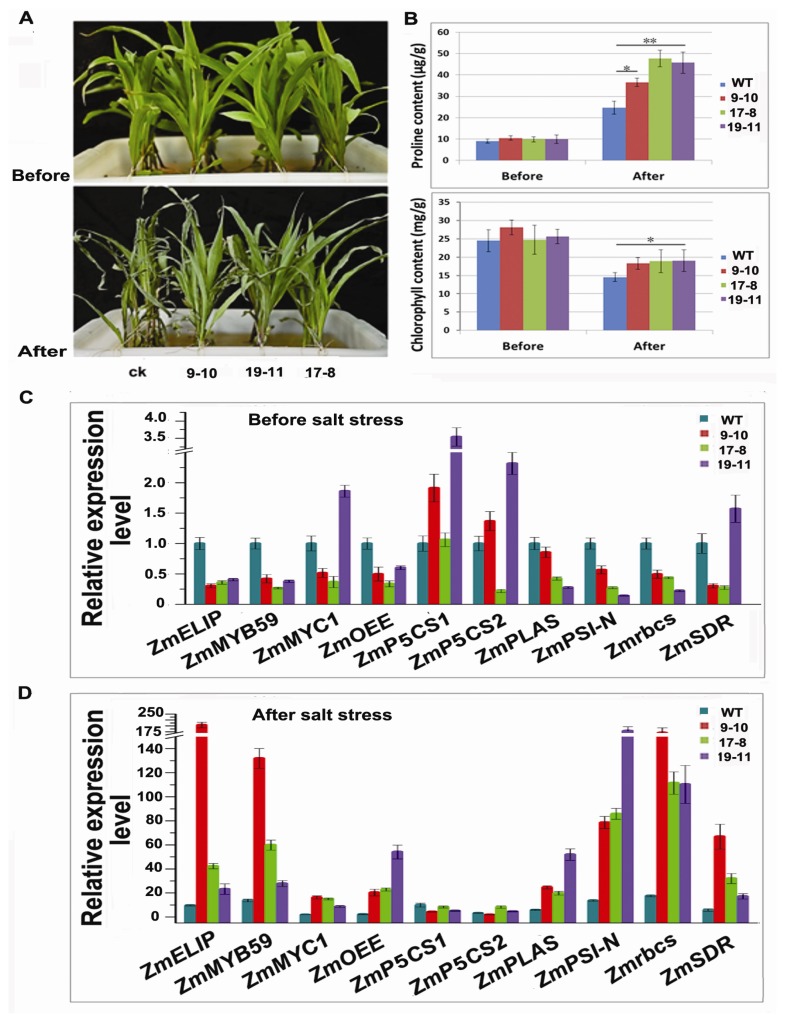
*TSRF1* improved salt tolerance in maize. (**A**) Salt tolerance of *TSRF1* transgenic maize. Before, control experiment without drought treatment; after, maize seedlings at four-leaf stage were subjected to 200 mM NaCl stress for 48 h; (**B**) proline and chlorophyll content in the control and transgenic maize. Data are averages of triplicate ± standard deviations. Asterisks, * and **, denote statistically different from wild-type by Student *t*-test at *p* < 0.05 and *p* < 0.01, respectively; (**C**) relative expression of stress-related genes in wild-type and *TSRF1* transgenic maize seedlings before salt treatment; (**D**) relative expression of stress-related genes in wild-type and *TSRF1* transgenic maize seedlings after salt treatment. Total RNA extracted from leaves of maize seedlings before and after 200 mM NaCl treatment for 48 h was used, respectively. *Tubulin* was used as a reference gene. Data are averages of triplicate ± standard deviations.

The overexpression of *TSRF1* increases pathogen tolerance in tobacco and tomato [29] and drought resistance in rice [32]. To investigate whether TSRF1 have salt tolerance in transgenic maize, four-leaf stage seedlings of three transgenic lines were used for salt treatment. The result was shown in Figure 5A. Before treatment, these transgenic lines displayed similar growth situation as non-transformed control, suggesting that the expression and insertion of *TSRF1* into maize genome did not influence the growth of transgenic lines. After treated in Hoagland solution containing 200 mM NaCl for 48 h, both non-transformed and transgenic plant leaves displayed wilting and losing green symptoms. However, the degree of control seedlings was much more serious than that of transgenic lines. All the leaves of control seedlings were wilting and rolling. However, the first and second new leaves were still flat and green in transgenic seedlings.

Proline was considered to be an osmoprotectant to protect plants against the damage resulting from abiotic stress [53]. Plants accumulate high level of proline under stress conditions to prevent the detrimental change. In addition, there is strong evidence that salt stress affects photosynthetic enzymes, chlorophyll and carotenoids in plants [54]. Therefore, we measured the free proline and chlorophyll content change in control and transgenic lines after salt treatment. As it is shown in Figure 5B, the free proline contents in control and transgenic seedlings were at similar level before treatment and increased dramatically after salt treatment. However, the proline content in the three transgenic lines increased at a much larger extent. Similarly, the chlorophyll content in transgenic seedlings decreased at a lower extent after salt treatment, indicating the more tolerance of *TSRF1* overexpressing transgenic maize.

It was previously shown that the constitutive expression of *TSRF1* enhanced rice salt and drought resistance by activating the expression of MYB, MYC, proline synthesis and photosynthesis-related genes [32]. Therefore, we further investigated whether TSRF1 affects the expression of these genes in maize. Ten genes were selected to be analyzed, and the results were shown in Figure 5C,D. The expressions of these genes were found to be different between transgenic lines. This might be due to the different transgenic lines with different target gene insertion sites and the expression levels of *TSRF1*. Under normal conditions, the expression level of *ZmMYC1* in line 19–11, *ZmP5CS1* (Δ^1^-pyrroline-5-carboxylate synthetase 1) and *ZmP5CS2* (Δ^1^-pyrroline-5-carboxylate synthetase 2) in line 9–10 and 19–11 and *ZmSDR* (short-chain dehydrogenase/reductase) in line 19–11 were increased, while the expression of these four genes in other lines were inhibited. The other six genes, including stress responsive transcription factor genes—*ZmMYB59*—photosynthesis-related genes—*ZmELIP* (low molecular mass early light-inducible protein HV90), *ZmPSI-N* (photosystem I reaction center subunit N), *ZmOEE* (photosystem II oxygen-evolving complex protein), *Zmrbcs* (encoding ribulose 1, 5-bisphosphate carboxylase small subunit) and *ZmPLAS* (encoding plastocyanin)—were inhibited in all these three transgenic lines, which was different from that in rice [32]. This indicated that TSRF1 was probably involved in different regulatory pathways in maize from that in rice. Furthermore, we detected the expression changes of these genes in wild-type and transgenic lines after salt stress. As is shown in Figure 5C, the expression of all these ten genes were increased by 2–16-fold in wild-type after salt treatment, indicating the endogenous regulation of salt responsive genes. Intriguingly, much more fold accumulations of these genes were induced after salt stress in *TSRF1* overexpressing lines. For example, the expression of *Zmrbcs* had increased as high as 171-, 111- and 110-fold in transgenic lines 9–10, 17–8 and 19–11, respectively, compared with wild-type. The sharp induction of photosynthesis-related genes (*ZmELIP*, *Zmrbcs* and *ZmPSI-N*) was consistent with the result of chlorophyll content change in Figure 5B. TSRF1 increases ABA sensitivity in tobacco [31,55] and rice [32]. *SDR*, an ABA biosynthesis related gene, was increased in *TSRF1* overexpressing rice [32]. On the contrary, it was slightly suppressed in line 9–10 and 17–8 under normal condition. After salt treatment, surprisingly, TSRF1 triggered significant *ZmSDR* induction. All these results suggested the complicated interaction of TSRF1 and ABA in plants. The accumulation of transcription factor gene *ZmMYB59* increased much more than *ZmMYC1* in all the transgenic lines, demonstrating the different roles of stress responsive transcription factors in salt stress. They may induce different other downstream salt related genes. These results indicated the complexity mechanism of *TSRF1* that involved in maize salt stress regulatory network.

Moreover, we noted that the expression levels of *ZmP5CS1* and *ZmP5CS2* were not or slightly increased in transgenic lines under salt condition, compared to wild-type, even though high level of proline accumulation was observed (Figure 5B,C). It is reported that the activity of P5CS, a key enzyme representing a rate-limiting step in proline biosynthesis, is regulated through feedback inhibition by proline [56–59]. It is possible that a large amount of proline is synthesized at the early stage of salt stress and maintained this high level throughout the salty condition. Therefore, high activity of P5CS or high level of *P5CS* transcript might be not necessary for high proline content maintaining under salt condition.

## 3. Experimental Section

### 3.1. Plasmid Construction

Diagrams of two plasmids used in this study were shown in Figure S1. The plasmid pTSRF1 containing the *TSRF1* expression cassette was kindly provided by Prof. Rongfeng Huang, Biotechnology Research Institute, China Academy of Agricultural Sciences (CAAS). The TSRF1 expression cassette was obtained by digesting pTSRF1 with *Bam*HI and *Hin*dIII, blunt-ended with Klenow enzyme (Promega, Madison, WI, USA) and then inserted into the blunt-ended *Hin*dIII site of p19SB, harboring the *SBgLR* expression cassette. The plasmid pHpt was constructed by Wang *et al.* 2006 [44].

### 3.2. Plant Transformation and Detection

Maize inbred line X178, the male parent of the leading commercial cultivar Nongda108 in China, was grown in the test field and was self-pollinated. Embryogenic calli were induced from immature embryos at 10–12 DAP and transformed by particle bombardment, as described by Yu *et al*. 2004 [13].

For transgene detection, genomic DNA was extracted from leaf of transformed plants using SDS method [60], and PCR amplification was performed as follows: 95 °C for 10 min, 30 cycles of 95 °C for 30 s, 54 °C for 30 s and 72 °C for 30 s.

The expression of *SBgLR* and *TSRF1* in transgenic plants was further determined by RT-PCR. Total RNA was extracted from immature seeds (22 DAP) and young leaves, respectively, using TRIzol reagent (Invitrogen, Carlsbad, CA, USA). The first strand of cDNA was synthesized according to the manufacturer’s protocol of Reverse Transcription System (Promega, Madison, WI, USA). *Tubulin* was used as the endogenous reference. PCR reactions were carried out for 5 min at 95 °C, followed by 25 cycles of 40 s at 94 °C, 40 s at 54 °C (*SBgLR* and *TSRF1*) or 52 °C (*Tubulin*), 40 s at 72 °C and a final extension of 10 min at 72 °C.

Genomic DNA dot blot analysis was used to detect marker-free progeny of T_2_ plants. Total genomic DNA was isolated from fresh leaves using the cetyl trimethyl ammonium bromide (CTAB) procedure [61]. A 10 μg aliquot of DNA was spotted onto nylon membrane (Amersham Pharmacia, Arlington Heights, IL, USA). Filters were hybridized using specific purified PCR fragments of *SBgLR* and *Hpt*, respectively, as described for Southern blot by Yu *et al*. 2004 [13]. The primer pair for Hpt gene was the same as reported [14]. The primer pairs for *SBgLR*, *TSRF1* and *Tubulin* were listed in Table S1.

The lysine-rich protein SBgLR accumulation in transgenic plant seeds was examined by Western blot, as described by Lang *et al.* 2004 [14]. Specific rabbit polyclonal SBgLR antiserum was prepared by the Institute of Genetics and Development Biology, Chinese Academy of Sciences (CAS).

### 3.3. Salt Stress Treatment

We determined the salt tolerance assay of transgenic maize at T_3_ generation. Seeds were sowed in nurse soil and grown to four-leaf stage at the condition of 28 °C and 16 h light/8 h dark. Before salt treatment, leaves of one half wide type and transgenic lines were collected and immediately stored in liquid nitrogen, respectively. The other half was used for subsequent free proline and chlorophyll content analysis. For salt tolerance assay, after washing the soil off the roots, the seedlings of wild-type and transgenic lines were treated in Hoagland solution with 200 mM NaCl for 48 h, respectively. As described above, leaves of one-half salt treated wild-type and transgenic seedlings were collected and immediately stored in liquid nitrogen, respectively. The other half was used for subsequent free proline and chlorophyll content analysis.

### 3.4. Detection of Free Proline and Chlorophyll Content

Free proline was extracted from 0.5 g maize leaves (dry weight) using 3% salicylsulfonic acid and then reacted with ninhydrin, followed by determination based on colorimetric method [62].

For chlorophyll a and b content detection, total chlorophyll was extracted from 0.5 g leaves (dry weight), grinded into homogenate in 95% ethanol, and then absorbance at the wavelength 649 and 665 nm was measured under a spectrophotometer (UV2300, Shanghai, China), respectively. The chlorophyll content of each sample was calculated using the equation as follows: *C*_a_ = 13.7*A*_665_ − 5.76*A*_649_; *C*_b_ = 25.8*A*_649_ − 7.6*A*_665_; *C*_a+b_ = *C*_a_ + *C*_b_.

### 3.5. Gene Expression Analysis by Quantitative Real-Time PCR

Total RNA of maize seedling leaves were extracted using TRIzol reagent (Invitrogen, Carlsbad, CA, USA). After digested with DNase I, 5 μg total RNA was used for cDNA first strand synthesis according to the manufacturer’s protocol of Reverse Transcription System (Promega A3500, Madison, WI, USA). For quantitative real-time PCR amplification, 100 ng cDNA was used as template in a 20 μL reaction system, containing 10 μL 2× SYBR Premix Ex Taq II (TaKaRa, Shiga, Japan), 0.5 μM each specific forward and reverse primer. Amplification was carried out on a Bio-Rad Real-Time System CFX96 C1000 Thermal Cycler (Bio-Rad, Hercules, CA, USA) using the following conditions: 95 °C for 30 s, 35 cycles of 95 °C for 10 s, 60 °C for 10 sec and 72 °C for 10 s. Four reference genes, including *Tubulin*, *EF1-α*, *GAPDH* and *Ubiquitin*, were chosen. Expression levels of these four genes were determined as the Ct values [63]. The Ct values were then converted into relative quantities and imported into the geNorm v3,5 software [64] for stability analysis. The result was shown in Figure S3. According to the result, *Tubulin* was selected to be used as the endogenous reference. All primer pairs used for qRT-PCR were listed in Table S1.

### 3.6. Crude Protein and Lysine Content Analysis

Samples were ground meal of 20 mature kernels from individual self-fertilized ears. Due to limited seeds, only 8 T_1_ mature kernels for line 19–11 were used. For crude protein content, total nitrogen was first measured based on the principle of Kjeldahl determination under national standard GB2905-82, Beijing Academy of Agriculture and Forestry Science. Then, the nitrogen content was converted to protein content by multiplying a conversion factor of 6.25.

Lysine content was analyzed based on the principle of ninhydrin reaction. Briefly, about 10 mg of defatted powder, 1 mL double distilled water and 2 mL ninhydrin reaction reagent were added into a 30 mL tube, and then the mixture was thoroughly vortexed (approximately 5 s). Subsequently, the tube was incubated in boiling water for 20 min, and thereafter, 3 mL 50% ethanol was added to cooled sample, followed by a centrifugation at 13,400× *g* for 10 min. The supernatant was collected, and the absorbance at 570 nm was recorded using a spectrophotometer (Techcomp UV2300, Shanghai, China). The standard curve was conducted by using 50 μg/mL leucine solutions in parallel. Lysine content of each sample was calculated using the equation as follows: Lys content (g/100 g dry seed weight) = (measured lysine content × hydrolysis volume)/sample weight.

## 4. Conclusions

In summary, we obtained marker-free transgenic maize inbred X178 lines harboring a potato lysine-rich protein gene, *SBgLR*, and a tomato ERF transcription factor gene, *TSRF1*, via particle bombardment mediated co-transformation. Molecular analysis revealed that both of these two target genes were successfully expressed and showed various expression levels in different transgenic lines. Quantification of protein and lysine content in T_1_ maize seeds showed that transgenic lines had both increased protein and lysine content. The protein and lysine content increased by 7.7% to 24.38% and 8.70% to 30.43%, compared to non-transformed maize, respectively. Moreover, transgenic maize exhibited more tolerance to salt stress. This study provides an effective way of corn molecular breeding and materials for improved nutritive quality and salt tolerance.

## Supplemental Information



## Figures and Tables

**Table 1:** Efficiency of particle bombardment-mediated maize co-transformation.

Ratio of pTSSB/pHpt	No. of transformed calli	No. of hygromycinresistant events recovered	No. of regenerated plants	PCR analysis	Co-transformation efficiency (%)

				*SBgLR* (+) *TSRF1* (+) *Hpt* (*+*)	
1.5:1	2411	224	114	26	1.08 a

a Co-transformation efficiency = (No. of all three genes PCR positive plants/No. of calli transformed) × 100%.
