# Supplementary material for: Improved Nutritive Quality and Salt Resistance in Transgenic Maize by Simultaneously Overexpression of a Natural Lysine-Rich Protein Gene, SBgLR, and an ERF Transcription Factor Gene, TSRF1
Source: Int J Mol Sci. 2013 Apr 29;14(5):9459–74. doi: 10.3390/ijms14059459 (PMC3676793; doi:10.3390/ijms14059459)
Supplement: Supplementary file 1 [file ijms-14-09459-s001.pdf]

Supplemental Information

Figure S1. Diagram of the two constructs used in the present study (not drawn to scale). (**A**) pTSSB; (**B**) pHpt. 35S Pro, *cauliflower mosaic virus* 35S promoter; 19Z Pro, maize 19 kD zein gene promoter; Adh1-int, the first intron of maize alcohol dehydrogenase gene 1; NOS-ter, nopaline synthase terminator; Hpt, hygromycin B phosphotransferase gene.

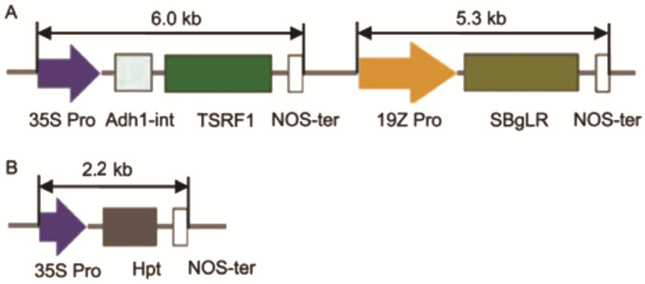

Figure S2. Transgenic plants derived from maize embryogenic calli by particle bombardment-mediated co-transformation. (**A**) Embryonic calli of maize inbred X178; (**B**) Hygromycin-resistant calli after 6 weeks of selection; (**C**) Shoot formation from the hygromycin-resistant calli. The photo was taken 3 weeks after hygromycin-resistant calli had been placed on hygromycin-containing regeneration medium; (**D**) Root development from hygromycin-resistant shoot after 2 weeks culture in the rooting medium; (**E**) Fertile plants in the greenhouse; (**F**) Seed set on the ears of R_0 plants; (**G**) PCR analysis of some R_0 plants for *SBgLR* gene. Lane 1: DNA Ladder, Lane 2: Positive control, Lane 3-8: Transformed plants, Lane 9: Untransformed plants as negative control; (**H**) PCR analysis of some R_0 plants for *TSRF1* gene. Lane 1: DNA Ladder, Lane 2: Positive control, Lane 3-9: Transformed plants, Lane 10: Untransformed plants as negative control. Scale bars: A–D and F, 1 cm; E, 30 cm.

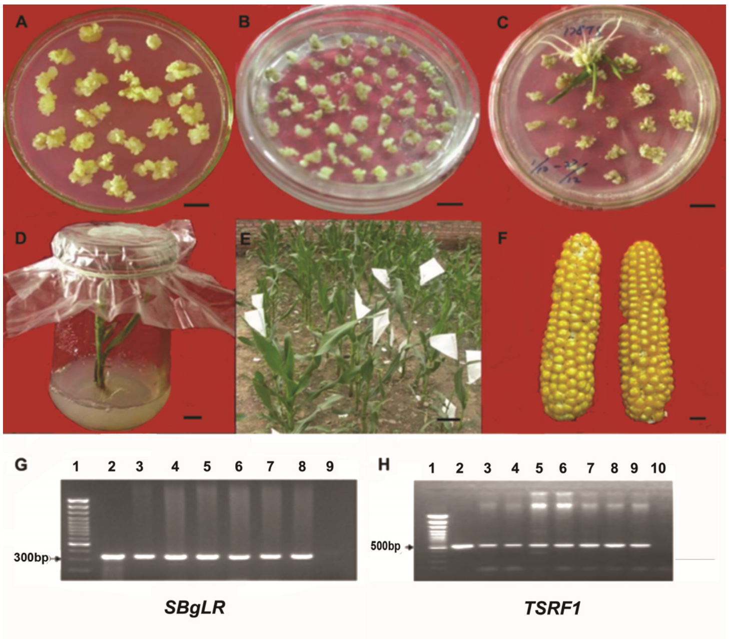

Figure S3. Average expression stability values of reference gene candidates by geNorm analysis.

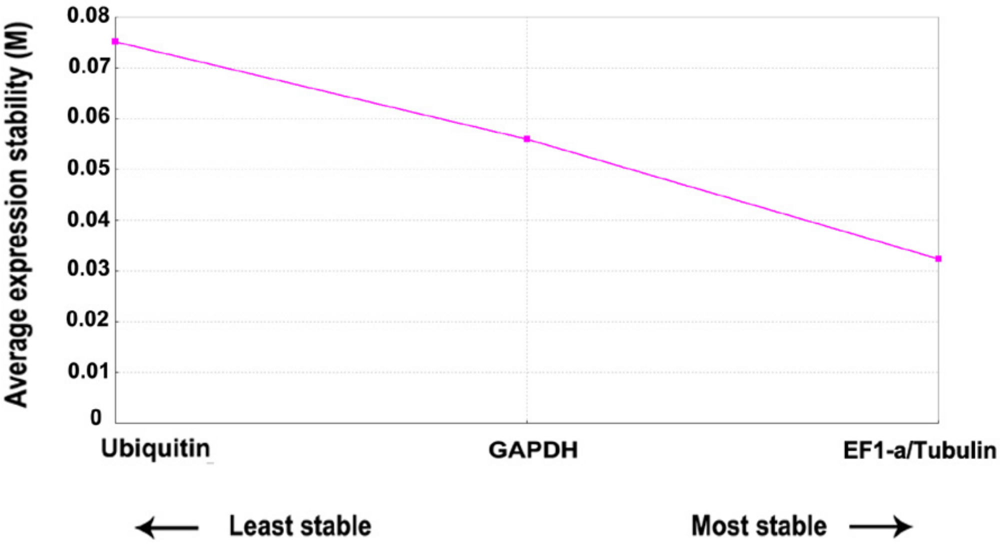

Table S1. Primers used in this study. The primers were designed using the Primer 3.0 software (version 0.4.0) (http://frodo.wi.mit.edu/). The accession numbers of these genes and the amplification lengths were described. Primer sequences of each gene for different uses were listed. F means forward primer. R means reverse primer.

Gene	Accession No.	Use	Primer sequences (forward and reverse)	Amplicon length (bp)
SBgLR	AY377987.1	PCR/Dot blot	F: 5'-TTGGAAATTCTCAACCTTGAA-3'	260
			R: 5'-ATGAAGAATTGGAGGAACTTGC-3'	
		RT-PCR	F: 5'-GAACGATGAAACAACTCCGGTT-3'	181
			R: 5'-GGCCTCAGAAACAGCGATAACTT-3'	
TSRF1	AF494201.1	PCR/RT-PCR	F: 5'-ACAACATCCGAAACAGTCAC-3'	472
			R: 5'-CAGCACCCAAATCTTCAAAC-3'	
Tubulin	NM_001174192.1	RT-PCR/qPCR	F: 5'-GAGCATGGCATTCAGGCTGTCG-3'	128
			R: 5′-TCAACAAAAACAGCACGGGGA-3'	
GAPDH	NM_001112119.1	qPCR	F: 5'-CCTTCCAGGGACTGAAAGACAG -3'	200
			R: 5'-CTCCAAATCTCACGTGGCTA -3'	
Ubiquitin	NM_001153555.1	qPCR	F: 5'-AGCTCCGACACCATCGACAA-3'	180
			R: 5'-TTACTGACCACCGCGGAG -3'	
EF1-α	NM_001112117.1	qPCR	F: 5'-GGTGATGCTGGTATGGTGAA-3'	192
			R: 5'-TCATTTCTTCTTGGCAGCAG-3'	
ZmMYC1	EU953366.1	qPCR	F: 5'-AATGAGCTGCGAGACGAGAAGCAA-3'	192
			R: 5'-AGGGTAGCCAATCACAGGCATCAT-3'	
ZmMBY59	CM000780.2	qPCR	F: 5'-TGCCATCACCTTCATCCTCATCCT-3'	190
			R: 5'-TGTGGTGCCTCAATCTCCTTCCAT-3'	
ZmSDR	GK000032.2	qPCR	F: 5'-TTGGAGCAGGCAATGGAGGGCAT-3'	178
			R: 5'-CTTTGGACGTTCCAGTGGATGGAT-3'	
ZmrbcS	Y09214.1	qPCR	F: 5'-GCAACAAGAAGTTCGAGACGCTGT-3'	130
			R: 5'-TACACGAACCCGAGCTTGCTGAA-3'	
ZmELIP	BT034060.1	qPCR	F: 5'-CCAACGCCGAGCTCTGGAA-3'	97
			R: 5'-TACGCGTTGATGAACGGTGCG-3'	
ZmPSI-N	BT034060.1	qPCR	F: 5'-CCAACAAGGAGCTGAACGACAAGA-3'	167
			R: 5'-AATCTCGAGGTCGTCGCTGATGAA-3'	
ZmOEE	CM000781.2	qPCR	F: 5'-GTACCAGATGAAGAAGCTGTGCCT-3'	192
			R: 5'-ATGCCGTCCTTCTCCTCGAACTT-3'	
ZmPLAS	NM_001154032.1	qPCR	F: 5'-AAGATCTCGCAGGAGGAGTACCTCA-3'	135
			R: 5'-TTAGTTGACGGTGATCTTGCCGAC-3'	
ZmP5CS1	BT083588.1	qPCR	F: 5'-ATCCTTGTGACCTCAGGTGCTGTT-3'	197
			R: 5'-AGTTGCGAGGAGGACACATCAAGT-3'	
ZmP5CS2	CM000784.2	qPCR	F: 5'-GCAAGTTGATAGTGCCGCTGTGTT-3'	195
			R: 5'-ACTCCCTTGTCACCATTCACCACT-3'	

Table S2. Lysine content of T_2 seeds. Samples were ground corn meal of 20 mature kernels from T_2 individual self-fertilized ears. The lysine contents are expressed as g/100 g dry seed. Data are averages of triplicate ± standard deviations. Asterisks ** denote transgenic lines statistically different from control by Student *t*-test at $p < 0.01$.

Line number	Lysine content (g/100 g dry weight)	Increase (%)
9-10	0.308 ± 0.06	48.79 **
9-11	0.284 ± 0.01	37.20 **
10-9	0.337 ± 0.01	62.80 **
12-8	0.318 ± 0.02	53.62 **
17-8	0.287 ± 0.01	38.65 **
19-11	0.306 ± 0.05	47.83 **
20-9	0.314 ± 0.02	51.69 **
21-9	0.243 ± 0.01	17.39 **
control	0.207 ± 0.02	-

© 2013 by the authors; licensee MDPI, Basel, Switzerland. This article is an open access article distributed under the terms and conditions of the Creative Commons Attribution license (http://creativecommons.org/licenses/by/3.0/).
